# Elevated EDAR signalling promotes mammary gland tumourigenesis with squamous metaplasia

**DOI:** 10.1038/s41388-021-01902-6

**Published:** 2021-12-16

**Authors:** Rebecca Williams, Stephanie Jobling, Andrew H. Sims, Chunyan Mou, Lorna Wilkinson, Giovanna M. Collu, Charles H. Streuli, Andrew P. Gilmore, Denis J. Headon, Keith Brennan

**Affiliations:** 1 Faculty of Biology, Medicine & Health, Manchester Academic Health Science Centre, University of Manchester, Manchester, UK; 2 Applied Bioinformatics of Cancer, Edinburgh Breakthrough Unit, Institute of Genetics and Molecular Medicine, Edinburgh Cancer Research Centre, Edinburgh, Midlothian UK; 3 The Roslin Institute and Royal (Dick) School of Veterinary Studies, University of Edinburgh, Midlothian, UK

**Keywords:** Breast cancer, Oncogenes, Growth factor signalling, Disease model, Transdifferentiation

## Abstract

Ectodysplasin A receptor (EDAR) is a death receptor in the Tumour Necrosis Factor Receptor (TNFR) superfamily with roles in the development of hair follicles, teeth and cutaneous glands. Here we report that human Oestrogen Receptor (ER) negative breast carcinomas which display squamous differentiation express *EDAR* strongly. Using a mouse model with a high *Edar* copy number, we show that elevated EDAR signalling results in a high incidence of mammary tumours in breeding female mice. These tumours resemble the *EDAR*-high human tumours in that they are characterised by a lack of oestrogen receptor expression, contain extensive squamous metaplasia, and display strong β-catenin transcriptional activity. In the mouse model, all of the tumours carry somatic deletions of the third exon of the *CTNNB1* gene that encodes β-catenin. Deletion of this exon yields unconstrained β-catenin signalling activity. We also demonstrate that β-catenin activity is required for transformed cell growth, showing that increased EDAR signalling creates an environment in which β-catenin activity can readily promote tumourigenesis. Together, this work identifies a novel death receptor oncogene in breast cancer, whose mechanism of transformation is based on the interaction between the WNT and Ectodysplasin A (EDA) pathways.

## Introduction

Ectodysplasin A receptor (EDAR) is one of eight death domain receptors encoded within the human genome [1]. During development, EDAR signalling is stimulated by its ligand, Ectodysplasin A (EDA), leading to signal transduction through EDAR-associated death domain to the canonical NFκB pathway. Rather than inducing apoptosis, as effected by other death domain receptors such as Tumour Necrosis Factor Receptor 1 (TNFR1) and FAS, this signal is essential for the formation of placodes, the first step in the development of many ectodermal appendages and glands, including hair, teeth and sweat glands [2]. In humans, loss-of function mutations within EDAR pathway components lead to Hypohydrotic Ectodermal Dysplasia (HED), which is characterised by missing or misshapen teeth, sparse hair, a reduced ability to sweat [3, 4] and lack of full mammary development [5, 6]. Similar phenotypes are seen in the mouse strains, *downless* (*Edar*^*dlJ*^) and *Tabby* (*Eda*^*Ta/Ta*^), which carry loss-of-function mutations in the receptor and ligand respectively [4, 7, 8], and when NFκB signalling is reduced in the developing skin [9]. The developmental action of EDAR is tightly associated with WNT/β-catenin signalling, with several levels of interaction between these pathways having been described in early hair follicle development [10, 11].

The role of EDAR signalling in tumour development has received little attention, despite its ability to activate NFκB [12] and the clear association of NFκB signalling with many different cancer types, including breast cancer [13–15]. Furthermore, its signalling in the skin induces the expression of known mammary gland oncogenes [11, 16, 17], including *Wnt10b* whose overexpression in the developing mammary gland induces precocious alveolar development and focal mammary adenocarcinomas [18]. Receptor Activator of Nuclear Factor κ B (RANK), a closely related member of the TNF receptor superfamily lacking a death domain, also contributes to progestin-induced mammary gland tumours during pregnancy [19].

Thus, based on its signalling function and its role in mammary development, we evaluated *EDAR* as a novel candidate mammary oncogene. We assessed expression data from human breast carcinomas, identifying a specific subtype of ductal carcinoma with squamous metaplasia that expressed *EDAR* strongly. To determine whether high EDAR levels are capable of causing breast cancer, we examined mammary tumour formation in a transgenic mouse line expressing high levels of *Edar* from its endogenous regulatory elements. We found that breeding female mice develop mammary tumours, and, as observed in the *EDAR*-high human tumours, these tumours contain extensive squamous metaplasia. Our analysis of the tumours indicated that the modestly increased EDAR signalling in the *Edar*^*Tg951/951*^ mice is further increased in transformed cells, as is β-catenin transcriptional activity. All tumours carry exon 3 deletions of *CTNNB1*, conferring WNT-independent activity on β-catenin. This β-catenin activity is required for tumour cell proliferation, highlighting the interplay between EDAR and WNT pathways in tumourigenesis. Together our data identify *EDAR* as a novel breast oncogene.

## Results

### *EDAR* is highly expressed in basal and metaplastic human breast cancers

**Fig. 1:**
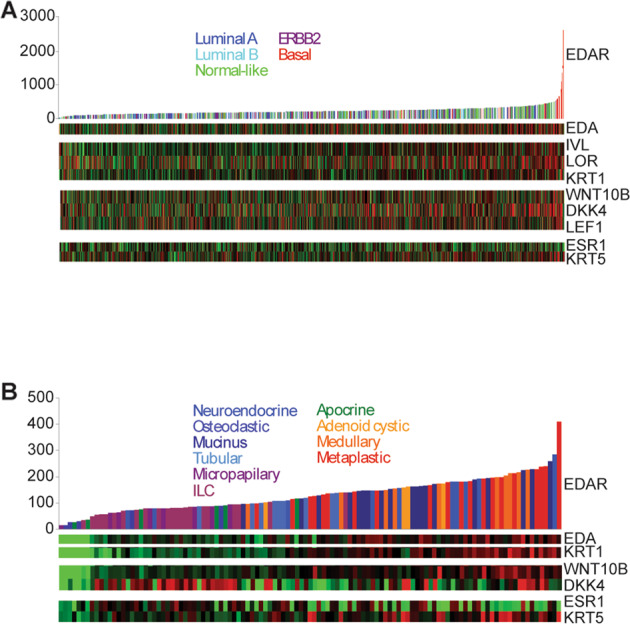
*EDAR* is highly expressed in basal and metaplastic human breast cancers. Expression of EDAR pathway components, skin markers and EDAR target genes in a meta-analysis of 1107 primary tumours (**A**), and 113 breast cancers of special histological type (**B**). Tumours were ordered left to right by increasing *EDAR* expression in both datasets and coloured by molecular (**A**) or histological subtype (**B**); white bars represent tumours that were not clearly assigned to any subtype. The heat bars show high expression (red) of *EDA*, skin markers (*IVL,KRT1*), and EDAR target genes, *WNT10B* and *DKK4* in *EDAR*-high tumours. Increased *LEF1* expression indicated increased WNT/β-catenin signalling in the *EDAR*-high tumours. Low expression (green) of *ESR* and high expression of *KRT5* was seen in the *EDAR*-high tumours confirming they were ER-ve tumours with a basal molecular subtype.

The action of EDAR in breast tissue, its similarity to the known mammary oncogene RANK, and its ability to activate NFκB, led us to investigate whether EDAR could be a causative factor in human breast cancer. We first analysed expression of *EDAR* in a meta-dataset of 1107 invasive human breast cancers classified into the basal-like, luminal A, luminal B, normal breast-like, and HER2 molecular subtypes [20, 21]. Within this dataset, there was a small group of tumours that expressed very high levels of *EDAR*, which almost invariably belonged to the basal-like subgroup (Fig. 1A). These basal-like *EDAR*-high tumours have low *ESR* expression, which encodes the oestrogen receptor. There was also a positive correlation between high *EDAR* and expression of the skin-specific genes *LORICRIN* (*LOR*), *KERATIN1* (*KRT1*) and *INVOLUCRIN* (*IVL*), suggesting the occurrence of squamous metaplasia within the *EDAR*-high cancers (Fig. 1A).

In light of the correlation between *EDAR* and the expression of epidermal marker genes, we also examined *EDAR* expression in a second dataset of 113 histologically special type tumours, which include metaplastic cancers that can display extensive squamous metaplasia (Fig. 1B) [22]. The tumours expressing the highest levels of *EDAR* were the adenocystic, medullary and metaplastic cancers, which are those most closely related to the basal molecular subtype of invasive breast cancers of no specific type [23, 24]. These tumours also showed high levels of *KRT1* and *IVL* expression, indicating squamous differentiation (Fig. 1B). Thus elevated *EDAR* expression is associated with squamous metaplasia in human breast cancers.

### High *Edar* copy number in the *Edar*^*Tg951*^ line leads to elevated expression and signalling in the mammary gland

**Fig. 2:**
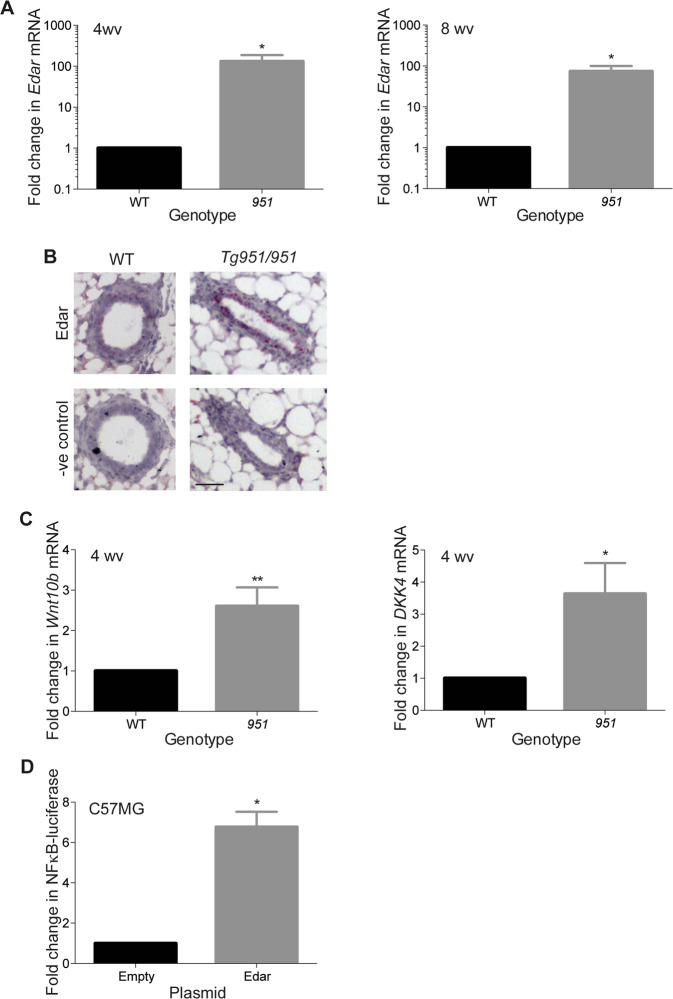
Increased *Edar* expression and function in the *Edar*^*Tg951*^ mouse line. **A** qRT-PCR analysis of *Edar* expression in nulliparous mouse mammary glands of wild-type (WT) and *Edar*^*Tg951*/*951*^ at 4 and 8 weeks of age (wv), normalised to *Keratin18* (**P < 0.01, *P < 0.05, *N* = 4). **B** In situ hybridisation to detect *Edar* and *dapB* negative control probesets on mammary gland sections from *Edar*^*Tg951/951*^ and WT female mice at 6 weeks of age. *Edar* expression was detected in the ductal luminal cells. Scale bar = 50 µm. **C** qRT-PCR analysis comparing expression of *Wnt10b* and *Dkk4* in WT versus normal *Edar*^*Tg951/951*^ mammary tissue at 4 weeks of age. Expression was normalised to *Keratin18*. *Wnt10b* and *Dkk4* expression were elevated in the *Edar*^*Tg951/951*^ mammary gland relative to WT (**P < 0.01, *P < 0.05, *N* ≥ 5). **D** Luciferase reporter assay detecting NFκB activity in C57MG cells in response to transfection of an *Edar* expression vector (*P < 0.05, N = 2). Error bars indicate SEM.

To assess whether elevated *Edar* expression can be a causative factor in mammary tumourigenesis, we employed the *Edar*^*Tg951*^ transgenic mouse line; homozygous *Edar*^*Tg951/951*^animals carry approximately 32 additional copies of the *Edar* locus [25]. We determined *Edar* expression levels in non-transgenic wild-type and homozygous *Edar*^*Tg951/951*^ mammary tissue at 4 and 8 weeks of age, finding increased *Edar* expression in the transgenic line (Fig. 2A). We assessed the location of *Edar* expression by in situ hybridisation, detecting the transcript in luminal cells of the epithelial ducts of both non-transgenic wild-type and *Edar*^*Tg951*/*951*^ mice (Fig. 2B). Increased *Edar* expression in the *Edar*^*Tg951/951*^ line is accompanied by elevated expression of the EDAR target genes *Wnt10b* and *Dickkopf4* (*Dkk4*) (Fig. 2C), demonstrating that signal transduction through this pathway is amplified in the transgenic mammary glands. To determine whether increased *Edar* expression can cause elevated signal transduction in mammary epithelial cells, we transfected C57MG mouse mammary epithelial cells with an *Edar* expression vector and assessed NFκB luciferase activity. Transfection and high-level *Edar* expression in this cell line did lead to increased NFκB activity (Fig. 2D) demonstrating the capacity of mammary epithelial cells to respond to high-level EDAR expression by activating NFκB.

### Elevated EDAR signalling leads to mammary tumourigenesis in breeding female mice

**Fig. 3:**
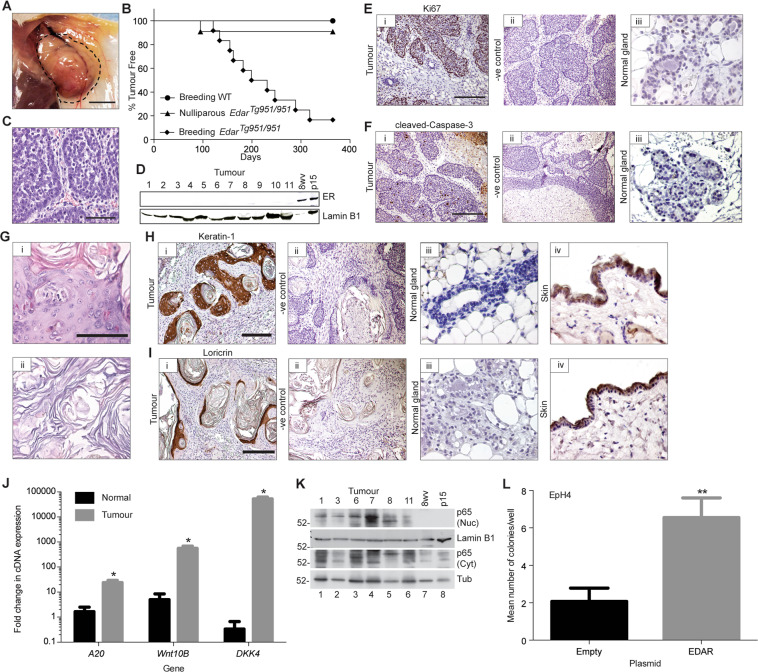
Elevated EDAR signalling leads to pregnancy-dependent mammary tumours with squamous metaplasia. **A** Gross image of a tumour from a breeding 6-month-old *Edar*^*Tg951/951*^ mouse. Dotted line shows the outline of the mammary tumour. Scale bar = 50 mm. **B** Mammary tumour incidence in continuously breeding and nulliparous cohorts of *Edar*^*Tg951/951*^ mice, compared to continuously breeding WT mice. Results are expressed as the percentage of mice remaining tumour free in cohorts of mice as a function of their age (median survival for breeding *Edar*^*Tg951/951*^ female mice 215 days, difference in survival curves *P* < 0.0001, *N* ≥ 10). **C** Histological section from an *Edar*-induced tumour showing the development of adenocarcinoma. Scale bar = 200 µm. **D** Western analysis of oestrogen receptor (ER) expression in nuclear protein lysates from *Edar*-induced mammary tumours. Lysates from 8 week nulliparous (8wv) and day 15 pregnant (p15) mammary glands were used as positive controls. Lamin B1 was used as a loading control. Lanes 1–11 represent lysates from 11 different *Edar*-induced tumours. Immunohistochemical analysis of Ki67 (**E**) and cleaved Caspase-3 (**F**) expression in *Edar*-induced mouse mammary tumours (i); in control sections with secondary antibody only (ii); and in normal mammary gland tissue lying adjacent to the tumour (iii). High levels of Ki67 and cleaved Caspase-3 are seen in the *Edar*-induced tumours, indicating that the tumours are highly proliferative and contain areas of necrosis. Scale bars = 800 µm in i–ii and 200 µm in iii. **G** Histological sections from *Edar*-induced mouse mammary tumours showing areas of (i) transdifferentiation to epidermis and (ii) associated Keratin-like pearls. Scale bar = 200 µm. Immunohistochemical analysis of KERATIN1 (**H**) and LORICRIN (**I**) expression in *Edar*-induced tumour sections (i); in control sections with secondary antibody only (ii); and in WT mammary gland tissue (iii) and skin (iv). KERATIN1 and LORICRIN mark the intermediate and terminally differentiated epidermal cells, respectively, and are absent in the WT mammary gland. Scale bar = 800 µm in i–ii, and 200 µm in iii–iv. **J** qRT-PCR analysis of *Edar* and EDAR target genes *A20*, *Wnt10b* and *Dkk4* in tumours relative to normal *Edar*^*Tg951/951*^ mammary tissue, normalised to *Keratin18* expression (*P < 0.05, *N* ≥ 3). **K** Western analysis of p65 expression in nuclear and cytoplasmic lysates from *Edar*-induced tumours and WT mammary glands from 8-week nulliparous (8wv) and day 15 pregnant (p15) mice. Lamin B1 was used as a loading control. Nuclear accumulation of p65 indicative of NFκB signalling was observed within the tumours. **L** Soft agar colony forming assay using EpH4 cells transduced with *Edar* expression vector (pCDH) (**P < 0.01, *N* = 3). Error bars indicate SEM.

Having defined increased *Edar* expression and signalling activity in the *Edar*^*Tg951*/*951*^ mammary gland, we next assessed the tumour incidence in these mice. We followed cohorts of female nulliparous and continuously breeding *Edar*^*Tg951/951*^, along with wild-type mice, over a 1-year period. The continuously breeding *Edar*^*Tg951/951*^ mice developed solitary mammary gland tumours from as early as 3 months of age and with an average latency of approximately 7 months (Fig. 3A, B). In contrast, no tumours occurred in the continuously breeding WT mice and only one of the nulliparous *Edar*^*Tg951/951*^ mice developed a tumour (Fig. 3B), as did a single male *Edar*^*Tg951/951*^ mouse in the colony. We also observed tumours in 2 out of the 3 *Edar*^*Tg951/+*^ breeding female mice that we have had in the colony. Histological analysis of the EDAR-induced tumours showed that all were adenocarcinomas (Fig. 3C). No tumours were noted in any other organ in the course of the study.

Analysis of clinical markers of human breast cancer revealed that the tumours arising in the *Edar*^*Tg951/951*^ mice lacked Oestrogen Receptor (ER) expression (Fig. 3D) were highly proliferative, with many cells staining positively for the proliferation marker Ki67 (Fig. 3E), and contained large areas of apoptosis, marked by cleaved Caspase-3 staining (Fig. 3F). All tumours also displayed areas of squamous metaplasia, albeit to varying extents, which in some cases led to the formation of Keratin-like pearls (Fig. 3G). This transdifferentiation into skin was verified by expression of KERATIN1 and LORICRIN, markers of intermediate and terminally differentiated layers of the skin, respectively (Figs. 3H, 3I). The areas of transdifferentiation also expressed KERATIN14, a marker of the basal layer of the skin, which is also expressed in myoepithelial cells within the normal mammary gland (Fig. S1). Tumour tissue displayed elevated EDAR target gene expression (Fig. 3J) and an increase in nuclear p65 (Fig. 3K), indicative of ongoing increased NFκB signalling.

To test whether elevation of EDAR signalling in epithelial cells of mature mammary glands is capable of causing transformation alone, we transduced EpH4 cells, a cell line derived from mammary epithelial cells taken from pregnant mice, with an *Edar* expressing lentiviral vector and determined colony number in a soft agar growth assay. Forced *Edar* expression led to a statistically significant increase in colony formation (Fig. 3L), demonstrating that short-term stimulation of the EDAR pathway is capable of transforming cells derived from the wild-type adult mammary gland.

### EDAR-induced tumours have high levels of β-catenin activity and carry somatic mutations in *CTNNB1*

**Fig. 4:**
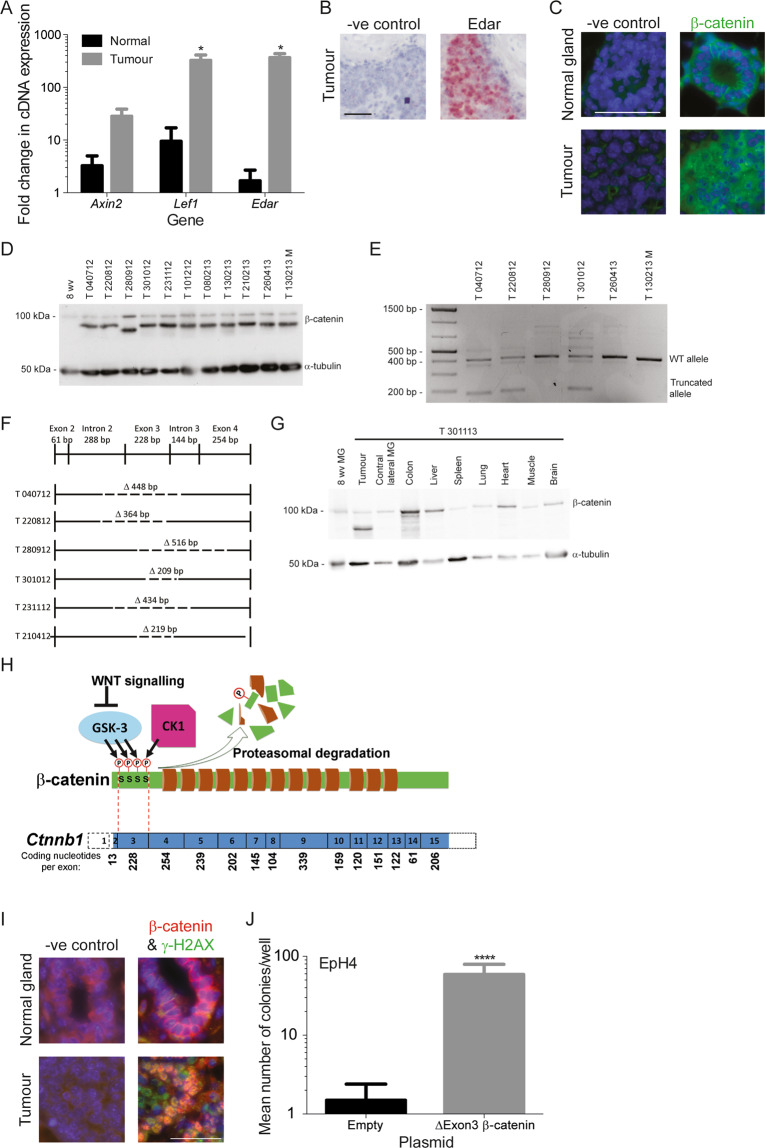
Edar-induced mammary tumours are characterised by high β-catenin activity. **A** qRT-PCR detection of WNT/β-catenin target genes *Axin2* and *Lef1* in tumours of *Edar*^*Tg951*/*951*^ mice (*P < 0.05, *N* ≥ 3). **B** In situ hybridisation detecting *Edar* expression in tumour epithelial cells. The *dapB* probeset is a negative control. Scale bar = 50 μm. **C** Immunodetection of nuclear β-catenin in tumours. Scale bar = 50 μm. **D** Western analysis of β-catenin protein in tumour homogenates. In addition to the full-length β-catenin, a more intense lower band representing a truncated form is detected in all tumour samples. PCR analysis across exon 3 of *Ctnnb1* showing products run on an agarose gel (**E**) and diagram of different mutations identified (**F**). **G** Western analysis of β-catenin protein in distinct tissues from the individual carrying tumour T301113. The short form of β-catenin protein is detected only in the mammary tumour isolate and not in other tissues. **H** Schematic of β-catenin gene and protein structure, with exon boundaries indicated by dotted lines and number of coding nucleotides per exon indicated below. Amino terminal phosphorylation sites, which induce protein degradation, are indicated by ‘P’. The function of the WNT signalling cascade, leading to suppression of GSK3 activity, is depicted. **I** Expression of γ-H2AX and nuclear β-catenin in normal *Edar*^*Tg951/951*^ gland and in tumour tissue. Scale bars = 50 μm. **J** Transformation of EpH4 cells by transduction with β-catenin expression constructs lacking the portion encoded by exon 3 (****P < 0.0001, *N* = 3). Error bars indicate SEM.

Based on the morphological similarities between *Edar*^*Tg951*/*951*^ mammary glands (See Supplementary material and Figs. S2–4) and those developing under conditions of elevated WNT signalling, and on the key role for WNT/β-catenin signalling as a driver of mammary tumorigenesis [26, 27], we assessed β-catenin status and function in *Edar*-induced tumours. qRT-PCR analysis identified elevated expression of β-catenin target genes *Axin2* and *Lef1* in *Edar*^*Tg951*/*951*^ tumour tissue (Fig. 4A), as well as elevated *Edar* transcript abundance when compared to untransformed *Edar*^*Tg951*/*951*^ mammary tissue (Figs. 4A, 4B). Nuclear immunostaining for β-catenin protein was also readily detectable (Fig. 4C), indicating its activated state. It is also interesting to note that within a series of mouse mammary tumour models elevated levels of *Edar* expression are only seen MMTV-Wnt1 mice (Fig. S5A).

We used Western analysis to assess β-catenin protein status in the tumour tissue. Surprisingly, in addition to the ~95 kDa band expected for full-length β-catenin, all tumours also displayed a smaller band with a more intense signal (Fig. 4D). These findings indicated that heterozygosity for a somatic mutation in *CTNNB1*, encoding β-catenin, could underlie the presence of a smaller form of this protein. We sequenced cDNA and genomic DNA from tumours, identifying in every case a mutation leading to deletion of *CTNNB1* exon 3; in one case the deletion encompassed part of exon 4 also (Fig. 4E, F). This shortened form of β-catenin is specific to tumour tissue and is not detected in other tissues, nor in the normal contralateral mammary gland (Fig. 4G and Fig. S5B). These somatic deletions remove the exon encoding the phosphorylation sites required for Glycogen Synthase Kinase 3 (GSK3)-triggered β-catenin degradation while maintaining the open reading frame, permitting WNT-independent β-catenin activity (Fig. 4H). Within the tumours, the nuclear β-catenin corresponded with sites of double-strand DNA breaks, as indicated by phospho-γ-H2AX staining (Fig. 4I). Repair of such double-strand breaks can occur through non-homologous end joining (NHEJ), which often leads to small deletion mutations consistent with those identified that remove the third exon of *CTNNB1*. The resulting exon 3 deleted forms of β-catenin selected in the tumours are sufficient to drive cellular transformation of EpH4 cells (Fig. 4J). Thus the high-level β-catenin activity observed in all *Edar*^*Tg951/951*^ tumours can be explained by their independent, but functionally identical, somatic mutations in *CTNNB1*.

### Involution and DNA damage in *Edar*^*Tg951/951*^ mammary glands

**Fig. 5:**
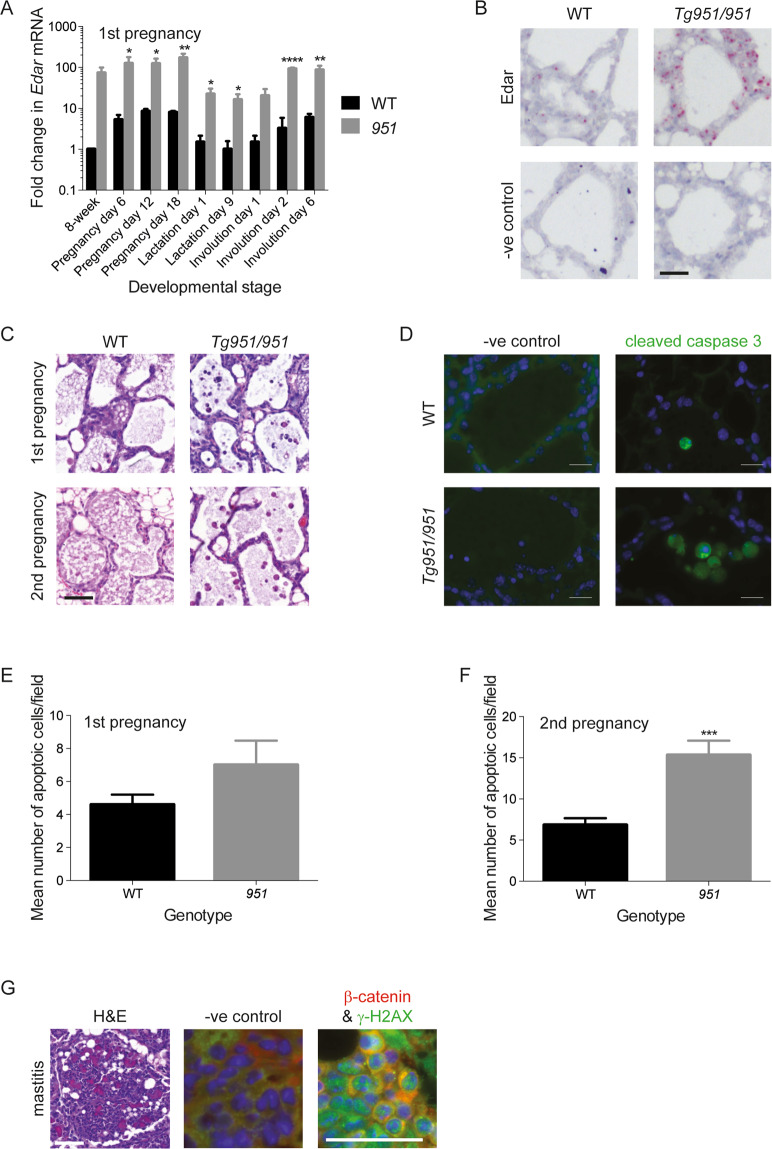
*Edar* expression and induced changes through the pregnancy/lactation cycle. **A** qRT-PCR analysis of *Edar* expression levels through the first pregnancy cycle in wild-type and *Edar*^*Tg951*/*951*^ glands (*P < 0.05, **P < 0.01, **P < 0.0001, *N* = 4). **B** In situ hybridisation detecting *Edar* expression in WT and *Edar*^*Tg951*/*951*^ mammary glands at involution day 2 (I2). Scale bar = 50 μm. **C** Histological assessment of mammary phenotype in *Edar*^*Tg951*/*951*^ mice at involution day 2 during first and second pregnancy. Scale bar = 50 μm. **D** Immunodetection of cleaved caspase-3 in mammary glands at involution day 2 during second pregnancy. Scale bar = 20 μm. **E**, **F** Quantification of apoptotic frequency in wild-type and *Edar*^*Tg951/951*^ mammary epithelia at involution day 2 (***P < 0.001, *N* ≥ 28). **G** Histological appearance of mastitis in *Edar*^*Tg951*/*951*^ gland found at involution day 2, accompanied by double-strand breaks identified as immunodetection of γ-H2AX. Scale bar = 50 μm.

Elevated EDAR signalling, together with activation of β-catenin activity through somatic mutation, appears to be a sufficient oncogenic stimulus to explain the high frequency of mammary tumours in the *Edar*^*Tg951/951*^ line. However, as it is not clear why continual breeding has such a marked effect on tumour incidence, we assessed *Edar* expression and function through the mammary gland’s pregnancy/lactation/involution cycle. qRT-PCR analysis detected *Edar* transcript at all stages of pregnancy, involution and lactation, with the lowest expression observed during lactation (Fig. 5A and Fig. S6A). In situ hybridisation shows that this expression occurs in luminal epithelial cells but is higher in *Edar*^*Tg951/951*^ than wild-type mammary glands (Fig. 5B and Fig. S6B).

Histological analysis of the mammary gland through pregnancy, lactation and involution reveals a similar structure in transgenic and non-transgenic at all stages (Fig. S6C), apart from early involution. At this stage, we observed an increased number of apoptotic cells in the *Edar*^*Tg951/951*^ mice (Fig. 5C–F), which was more pronounced after the second pregnancy than the first. However, as the area of epithelial tissue remaining in wild-type and *Edar*^*tg951/951*^ mice was not different at involution day 6 (Fig. S6D, E), we conclude that the increase in the number of apoptotic cells seen at involution day 2 in *Edar*^*Tg951/951*^ mice arose from a failure to clear apoptotic cells rather than an increased rate of apoptosis. We also noted a high rate of mastitis in breeding female *Edar*^*Tg951/951*^ animals (Fig. 5G) that we did not observe in wild-type mice. Mastitis may have arisen when milk ducts and acini became blocked with uncleared apoptotic cells in the transgenic line. Damaged DNA was also detected in mammary epithelial cells as γ-H2AX positive foci, revealing the presence of double-stranded DNA breaks (Fig. 5G). Thus the mastitis observed during mammary involution may provide an environment in which deletion mutations can more readily occur, potentially contributing to the mammary tumour incidence in the *Edar*^*Tg951*/*951*^ line.

### β-catenin activity is required for rapid proliferation of *Edar*^*Tg951*/*951*^ tumour cells

The morphological phenotype of the *Edar*^*Tg951/951*^ mammary gland is similar in most respects to that obtained by augmentation of WNT signalling [26, 27], and the activation of *Wnt10b* gene expression by increased *Edar* in transgenic mammary glands suggests a positive relationship between these pathways. However, if EDAR signalling had only a simple and positive relationship with β-catenin activity, then it is difficult to explain the observed selection for somatic mutations in *CTNNB1* that produce an activated β-catenin protein in tumours of the *Edar*^*Tg951/951*^ line. To address their signalling relationship, we assessed the short-term effects of EDAR signalling on β-catenin transcriptional regulatory activity by transfecting HEK293T cells with a β-catenin responsive luciferase reporter (pTOP-FLASH), together with an *Edar* expression construct. We found that introduction of high EDAR signalling in these conditions suppressed β-catenin activity (Fig. S7A). The transfection of a delEx3β-catenin expressing construct, which misses the portion of the protein encoded by the third exon and thus matches the truncated form observed in all *Edar*^*Tg951/951*^ tumours, evaded this EDAR driven inhibition and restored reporter gene response in transfected cells (Fig. S7A).

**Fig. 6:**
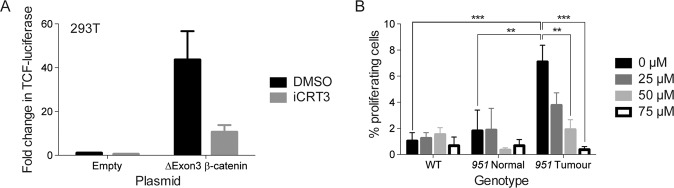
The elevated growth rate of EDAR transformed cells is dependent on β-catenin activity. **A** Luciferase assay detecting β-catenin reporter activity in cells transfected with delEx3β-catenin encoding constructs, with or without incorporation of iCRT3 to the culture (**P < 0.01, ***P < 0.001, *N* = 2). **B** EdU incorporation by cells from *Edar*^*Tg951/951*^ and wild-type mammary glands, and from *Edar*^*Tg951/951*^ tumours, grown in culture. iCRT3 administration suppresses proliferation of *Edar*^*Tg951/951*^ mammary tumour cells (**P < 0.01, ***P < 0.001, *N* = 3).

To assess the functional requirement for unrestrained β-catenin activity in *Edar*^*Tg951/951*^ tumour cell proliferation, we set out to impair β-catenin activity in these cells. Cells transfected with delEx3β-catenin display increased levels of TOPFLASH reporter activity, which is suppressed using iCRT3, a small molecule inhibitor of β-catenin interaction with TCF class transcription factors, validating its inhibitory action in this signalling context (Fig. 6A). We isolated mammary gland epithelial cells from wild-type, normal *Edar*^*Tg951/951*^ glands, and from *Edar*^*Tg951/951*^ tumours, and grew these in culture to assess their proliferative characteristics. Tumour cells incorporated the DNA synthesis label EdU at far greater frequency than normal cells, indicating their elevated rate of proliferation. Addition of iCRT3 to the culture medium suppressed proliferation of the tumour cells to levels that were not different from untransformed cells (Fig. 6B), demonstrating that β-catenin activity is required for elevated proliferative index of cells of the *Edar*^*Tg951/951*^ tumours; we also observed a decrease in the expression of the Wnt target gene *Lef1* in the iCRT3-treated tumour cells (Fig. S7B) but no change in the rate of apoptosis (Fig. S7C).

## Discussion

*EDAR* represented a potential mammary gland oncogene based on its signalling mode [28] and its role in mammary gland development [8, 29]. Our analyses of expression data from human breast cancer tissue identified a specific subtype of human breast cancer characterised by high *EDAR* expression. These tumours also displayed high expression levels of epidermal differentiation genes (such as *IVL*, *LOR* and *KRT1*), indicating that these tumours had undergone squamous metaplasia, and an activation of the WNT/β-catenin signalling pathway (expression of *WNT10B and LEF1*). The latter could explain the overexpression of *WNT10B* that is seen in some human breast cancers [30].

To test whether elevated EDAR signalling is sufficient to lead to mammary tumourigenesis, we employed the *Edar*^*Tg951*^ mouse model which carries a high copy number of *Edar* expressed from its endogenous regulatory elements [7, 25, 31]. This line has an altered rate of mammary gland development, such that ductal extension and branching is greater in the transgenic during puberty, but this morphological phenomenon is largely corrected by adult life. The high rate of mammary tumours in *Edar*^*Tg951*^ mice upon continual mating, which was not observed in wild-type mated or transgenic nulliparous females, demonstrated a driving role for EDAR as an oncogene. In this mouse model, the characteristic squamous metaplasia and expression of epidermal differentiation genes in mammary tumours matches the expression phenotype of the human metaplastic tumours with high *EDAR* expression. Thus in both human and mouse increased EDAR expression is associated with this distinct mammary tumour type.

Previous work has highlighted a role for NFκB signalling in mammary gland development and tumourigenesis. In particular, RANK signalling has been shown to play an essential role downstream of Progesterone Receptor in the formation of the milk-producing alveoli during pregnancy [32, 33] and to contribute to Progestin-induced mammary gland tumours [19, 34]. However there is some indication within the published literature that NFκB signalling may play a role in pubertal development as well. For example, transplanted mammary tissue, which lacks IκBα, forms outgrowths in the cleared fat pad of a nulliparous animal or matrigel that are substantially more branched than those derived from wild-type tissue [35]. These phenotypes have been attributed to changes in signalling activated by RANK. However given our results here and those of Voutilainen et al. [8], the phenotypes seen could instead be attributed to changes in EDAR-induced NFκB signalling. In fact, the extra branching seen in the ductal tree of virgin animals when RANKL is overexpressed from the mouse mammary tumour virus-long terminal repeat (MMTV-LTR) could be due to RANK/NFκB signalling mimicking endogenous EDAR/NFκB signalling at this stage [36]. However, we do not observe the precocious development of alveoli in the *Edar*^*Tg951/951*^ mice that is seen in the *MMTV-RankL* mice or the ductal hyperplasia observed in the transplanted tissue that lacks *IκBα* [35, 36]. This most likely reflects differences in the downstream targets of RANK and EDAR signalling. Mammary tumourigenesis has also been reported in the mouse models where RANK/NFκB signalling is activated within the mammary gland [19, 37]. Interestingly, most of the tumours that develop in the *MMTV-c-rel* mouse strain display squamous metaplasia [37], suggesting that WNT/β-catenin signalling may be activated in these tumours as well.

During development, EDAR signalling is intertwined with that of the WNT/β-catenin pathway [10, 11]. Both positive and negative interactions have been reported and suggested, based on target genes of each pathway. The morphological effect of increased EDAR function on the *Edar*^*Tg951*^ gland is consistent with an increase in WNT signalling [18, 38], and is consistent with increased *Wnt10b* expression, suggesting a stimulatory effect of EDAR on WNT pathway signalling over a sustained period. However, an inhibitory effect of EDAR signalling on β-catenin function is revealed by EDAR overexpression over a 48 h period in cultured cells, potentially through the activation of WNT/β-catenin pathway inhibitors such as *DKK4* [10, 11]. Thus, positive and negative impacts of EDAR function on β-catenin signalling are apparent. The negative impact of EDAR overexpression on β-catenin signalling may explain the absence in *Edar*^*Tg951/951*^ females of one mammary gland phenotype associated with excess WNT/β-catenin signalling, precocious lobular-alveloar development. This phenotype is seen when the pathway is activated directly by overexpressing a WNT protein or a non-degradable form of β-catenin [39, 40]. In these animals, β-catenin signalling is unregulated. In contrast, the expression of DKK4 or other negative regulators of WNT signalling will limit or control WNT/β-catenin signalling in the *Edar*^*Tg951/951*^ mice. If lobular-alveolar development requires a strong WNT/β-catenin signal, then it is reasonable to hypothesise that the signal is simply insufficient in *Edar*^*Tg951/951*^ females to drive lobular-alveolar development.

The short-term inhibitory relationship between EDAR and β-catenin is also likely to apply a selective pressure for unrestrained β-catenin activity to drive cell transformation. In tumours, the release of β-catenin activity from this inhibitory EDAR influence is provided by somatic mutation, with cells carrying β-catenin exon 3 deletions growing out to form tumours. The signalling environment caused by EDAR, with very high NFκB activity, likely provides a suitable environment for cells carrying these specific mutations to proliferate rapidly. The occurrence of deletion mutations can be explained by the high frequency of double-strand breaks in the EDAR driven tumours. In non-replicative cells, like the involuting mammary gland, these breaks are repaired by NHEJ which frequently leads to deletions like those observed. It is also interesting to note that mutations in WNT pathway components, although rare in human breast cancer in general, are common in metaplastic breast cancers [41].

The basis for the specificity of squamous metaplasia observed in EDAR driven tumours is unclear. EDAR acts during development of the surface ectoderm to control the patterning and morphogenesis of cutaneous appendages, but does not appear to have a direct influence on epidermal differentiation. Rather, EDAR signalling tends to drive cells away from an epidermal fate and towards that of ectodermal appendage [42]. Thus, it is unexpected, based on its developmental role, to find EDAR promoting growth of a differentiated epidermis within the mammary gland. Alternatively, the squamous metaplasia may simply reflect the operation of EDAR signalling outside its normal developmental context, particularly its operation in conjunction with WNT signalling. In this latter scenario, it is important to remember that squamous metaplasia is seen in many mouse models with unregulated Wnt/β-catenin signalling [26, 27].

Alleles of *EDAR* encoding a receptor with altered signalling potentials exist in human populations, with East Asian and Native American groups having a non-synonymous polymorphism leading to substantially increased signalling [25, 43]. These alleles may increase the incidence or progression of breast tumours with squamous metaplasia in these populations. A number of recombinant protein and antibody reagents to stimulate and repress the EDAR pathway in vivo have been developed recently [44, 45], with one of the stimulators in clinical trial for early-life correction of HED caused by EDA loss of function [46]. Such agents may represent useful modalities for future diagnosis and treatment of breast cancer with squamous metaplasia.

## Materials and methods

### Meta-analyses of microarray datasets

A meta-analysis of six Affymetrix gene expression datasets comprising 1107 primary human breast cancers was performed as previously described [21]. Centroid prediction [47] was used to assign the tumours to the five Norway/Stanford subtypes (Basal, Luminal A, Luminal B, ERBB2, and Normal-like) [48]. The dataset of 113 breast tumours representing special histological types [22] was downloaded from Array Express (http://www.ebi.ac.uk/arrayexpress/) experiment number E-NCMF-3. Treeview was used to generate heatmaps (http://rana.lbl.gov/EisenSoftware.htm).

### Animals

*Edar*^*Tg951/951*^ mice were on an inbred FVB/N genetic background and have been described previously [25]. All procedures carried out under the UK Home Office license. Cohorts of ten nulliparous or constantly breeding *Edar*^*Tg951/951*^ and wild-type FVB/N mice were established to analyse tumour formation. For the phenotypic analysis at least three animals were examined for each genotype and stage, specific numbers used are given in the appropriate figure legends. Randomisation and blinding were not used.

### Mammary gland whole mount preparation and morphological analysis

Whole mounts of the rostral inguinal (4th) mammary gland were prepared as previously reported [29]. Images of the whole mounts were captured digitally and morphometric analysis was performed using Image J 1.43 software.

### Immunohistochemistry

Rostral inguinal mammary glands and shaven back skin were fixed in 4% paraformaldehyde and paraffin-embedded. Antigen retrieval was carried out on mounted sections using a Borg Decloaker (Biocare Medical) and the sections were subsequently stained as described before [42]. Controls were performed using the secondary antibody only. See Supplementary Table 1 for the primary antibodies.

### In situ hybridisation

In situ hybridisation was carried out on 8-µm sections from paraffin-embedded mammary tissue using the RNAScope system (Advanced Cell Diagnostics, Hayward, CA), according to the manufacturer’s instructions.

### Western blotting

Cell lysates were obtained from powdered frozen mammary tissue samples in 1 ml 2× RIPA lysis buffer (300 mM NaCl, 100 mM Tris pH 7.4, 10 mM EDTA, 2% NP-40, 2% Deoxycholic acid, 0.2% SDS, 10 mM NaF, 1 mM NaVO_4_ and 1× Protease inhibitor cocktail (Calbiochem)) per gram of tissue and incubating for 15 min on ice. Lysates were then spun at 18,000 g to remove nuclei and genomic DNA. Nuclear-enriched fractions were obtained from powdered frozen mammary tissue samples in 1 ml NP-40 lysis buffer (10% w/v glycerol, 50 mM Tris-HCl pH 7.4, 100 mM NaCl, 1% v/v NP-40, 2 mM MgCl_2_, with protease/phosphatase inhibitors as above) per gram of tissue and incubating for 15 minutes on ice. Lysates were then spun at 18,000 g for 30 min at 4 °C. The nuclear pellets were resuspended in SDS buffer (10 mM Tris-HCl pH 7.4, 2% SDS) and genomic DNA was sheared using a probe sonicator. Equal quantities of the protein samples were run on SDS/polyacrylamide gels as described [49]. See supplementary Table 1 for all primary antibodies.

### Quantitative RT-PCR

RNA was extracted from mammary gland tissue using Trizol (Invitrogen) and treated with RNAse-free DNAse 1 to remove contaminating genomic DNA (New England Biolabs). Two micrograms of total RNA was reverse transcribed using the High Capacity RNA to cDNA kit (Applied Biosystems). The resultant cDNA was diluted tenfold and 2 µl was used as template for subsequent quantitative PCR reactions.

qPCR was carried out in triplicate using the primers listed in supplementary Table 2, the Step One Plus RT-PCR system (Applied Biosystems), and 2× SYBR-green PCR master mix (Applied Biosystems). PCR products were separated on 2% agarose gels to verify their size and the absence of non-specific products. The relative amounts of products were evaluated using the comparative Ct method, and *Keratin18* as a normalisation control to account for differences in the amount of ductal epithelium in each sample.

### Sequencing

Changes within the sequence of exon 3 of the β-catenin cDNA and genomic locus were screened and sequenced by amplifying the relevant region from either cDNA or the genomic DNA found within the initial RNA extraction prepared from tumours respectively, using the relevant primers listed in supplementary table 2. The amplified bands were then sequenced using nested sequencing primers (supplementary Table 2) using Big Dye Terminator (Applied Biosystems).

### FACS analysis

The cell populations present within the adult mammary gland were analysed as described in [50].

### Cell lines

The normal mouse mammary epithelial cell lines C57MG and EpH4 were gifts from Dr. Anthony Brown (Weill Medical College of Cornell University, USA) and Prof. Christine Watson (University of Cambridge, UK). Luciferase assays were conducted in HEK293T and EpH4 cells as described in [51], using *pNFĸB-Luc* and *pTopFlash* to monitor NFκB and WNT signalling respectively. EpH4 cells stably transduced with a *pCDH EF1α-Edar-T2A-GFP* or a *pCDH EF1α-ΔExon3-β-catenin-T2A-RFP* lentivirus were generated as described in [52]. Expression of the proteins was confirmed by western blotting and the expression of downstream target genes confirmed signalling (Fig. S8). Soft agar assays were carried out as described in [49].

### Statistical analyses

All data are presented as the mean ± SEM. Statistical significance was evaluated, where appropriate, using unpaired *t*-tests, one-way ANOVA followed by a Tukey’s test, or log-rank (Mantel–Cox) test.

## Supplementary information

Supplemental material

Supplementary Figure 1

Supplementary Figure 2

Supplementary Figure 3

Supplementary Figure 4

Supplementary Figure 5

Supplementary Figure 6

Supplementary Figure 7

Supplementary Figure 8
