# Supplementary material for: Elevated EDAR signalling promotes mammary gland tumourigenesis with squamous metaplasia
Source: Oncogene. 2021 Dec 16;41(7):1040–9. doi: 10.1038/s41388-021-01902-6 (PMC8837535; doi:10.1038/s41388-021-01902-6)
Supplement: Supplementary file 1 — Supplemental material [file 41388_2021_1902_MOESM1_ESM.doc]

**Supplementary material**

**Elevated EDAR signalling leads to phenotypes in the pubertal mammary gland that are reminiscent of those seen in male and female mice with elevated Wnt/β-catenin signalling**

At the morphological level, whole mount analysis indicated that the elevated EDAR signalling seen in *Edar^Tg951/951^* mice accelerated ductal outgrowth, leading to a greater area of the mammary fat pad containing ducts at 4 and 8 weeks of age (Fig. S2A, S2B ^2^), and increased ductal branching, resulting in many more ductal tips (Fig. S2E, S2F ^2^). Epithelial cells of the transgenic mammary gland also displayed elevated proliferative indices (Fig. S2C and S2D). However, by 12 weeks of age ductal outgrowth was not overtly different between *Edar^Tg951/951^* and wild type mice, demonstrating correction of this phenotype by maturity. Flow cytometric analyses revealed a shift towards myoepithelial and stem cell fates in the *Edar^Tg951/951^* gland (Fig. S3A, S3B). Histological analysis of the mammary gland revealed a normal bilayered epithelial architecture to the ducts of transgenic animals, with appropriate partitioning of KERATIN14 expressing myoepithelial cells and KERATIN18 positive luminal cells (Fig. S3C, S3D). The elevated EDAR signalling in male *Edar^Tg951/951^* mice resulted in the formation of an elaborately branched ductal tree structure, complete with terminal end buds, contrasting with the normally rudimentary mammary tree of wild type male mice (Fig. S4A). A similar ductal outgrowth phenotype has been noted in *Keratin14-EdaA1* transgenic male mice ^6^. We noticed the development of extra nipples along the milk line in *Edar^Tg951/951^* female mice (Fig. S4B). However, unlike the ectopic nipples seen in *Keratin14*-*EdaA1* mice ^5^, these ectopic nipples were associated with a very small mammary fat pad and showed negligible ductal development. We suspect that the difference in gland development associated with ectopic nipples in the two models reflects how Edar signalling is activated. In *Edar^Tg951/951^* mice, Edar signalling is activated in the tissues where Edar is normally expressed. In contrast, the expression of EdaA1 from a heterologous promoter in *Keratin14-EdaA1* mice will activate Edar signalling in novel tissues and at different developmental stages. Interestingly, considering the multiple links between Edar and Wnt signalling ^3, 7^, all of these pubertal phenotypes are all seen in mice with elevated Wnt/β-catenin signalling within the mammary gland ^1, 4^.

**Supplementary References**

1 Brennan KR, Brown AM. Wnt proteins in mammary development and cancer. J Mammary Gland Biol Neoplasia 2004; 9: 119-131.

2 Chang SH, Jobling S, Brennan K, Headon DJ. Enhanced Edar signalling has pleiotropic effects on craniofacial and cutaneous glands. PLoS One 2009; 4: e7591.

3 Fliniaux I, Mikkola ML, Lefebvre S, Thesleff I. Identification of dkk4 as a target of Eda-A1/Edar pathway reveals an unexpected role of ectodysplasin as inhibitor of Wnt signalling in ectodermal placodes. Dev Biol 2008; 320: 60-71.

4 Miyoshi K, Rosner A, Nozawa M, Byrd C, Morgan F, Landesman-Bollag E *et al*. Activation of different Wnt/beta-catenin signaling components in mammary epithelium induces transdifferentiation and the formation of pilar tumors. Oncogene 2002; 21: 5548-5556.

5 Mustonen T, Pispa J, Mikkola ML, Pummila M, Kangas AT, Pakkasjarvi L *et al*. Stimulation of ectodermal organ development by Ectodysplasin-A1. Dev Biol 2003; 259: 123-136.

6 Voutilainen M, Lindfors PH, Lefebvre S, Ahtiainen L, Fliniaux I, Rysti E *et al*. Ectodysplasin regulates hormone-independent mammary ductal morphogenesis via NF-kappaB. Proc Natl Acad Sci U S A 2012; 109: 5744-5749.

7 Zhang Y, Tomann P, Andl T, Gallant NM, Huelsken J, Jerchow B *et al*. Reciprocal requirements for EDA/EDAR/NF-kappaB and Wnt/beta-catenin signaling pathways in hair follicle induction. Dev Cell 2009; 17: 49-61.

**Supplementary Figure Legends**

**Supplementary Figure 1. KERATIN14 expression in *Edar^Tg951^*/*^951^* mammary tumours.**

Widespread KERATIN14 immunoreactivity is detected in tumours, while normal mammary gland and skin display expression in the basal layer of epithelium only. Scale bar = 800 µm in i-ii, and 200 µm in iii-iv.

**Supplementary Figure 2. Elevated EDAR signalling accelerates ductal outgrowth and branching in the pubertal mammary gland in female mice**

**A)** Whole mount mammary glands from female WT and *Edar^Tg951/951^* mice at 4, 8 and 12 weeks of age. Although EDAR signalling accelerated ductal penetration of the fat pad, the whole fat pad was filled in each genotype at 12 weeks. Scale bar = 5 mm. **B)** Quantification of ductal outgrowth into the fat pad at 4 & 8 weeks (* *P* <0.05, N=4). **C)** Ki67 staining of wild type and *Edar^Tg951/951^* mammary epithelium at 6 weeks of age. Scale bar = 100 μm. **D)** Quantification of proliferation in mammary glands at 6 weeks of age (* *P* <0.05, N=9). **E)** High magnification images of whole mount glands from female WT and *Edar^Tg951/951^* mice at 4 weeks of age. Ductal branching increased in *Edar^Tg951/951^* mice. Scale bar = 1 mm. **F)** Quantification of ductal branching at 4 weeks of age (**** *P* <0.0001, N=4).

**Supplementary Figure 3. Cell proportions and structure of wild type and *Edar^Tg951/951^* mammary glands.**

**A)** FACS scatterplot and **B)** quantitative analysis of cell types in WT and *Edar^Tg951^*/*^951^* glands. An increase in the proportion of stem/myoepithelial/basal cells is observed in the transgenic, identified by expression status of EpCAM and CD49f (* *P* <0.05, N=3). **C)** Histological sections of mammary glands from WT and *Edar^Tg951/951^* transgenic mice. Sections show the normal 2-layered epithelial structure with haemotoxylin and eosin staining and **D)** appropriate partitioning of KERATIN14 positive basal cells from KERATIN18 positive luminal cells in the *Edar^Tg951/951^* mammary gland. Scale bar = 100 µm in C and 50 μm in D.

**Supplementary Figure 4. Male mammary ductal structure and female nipple number in wild type and *Edar^Tg951^*/*^951^*mice.**

**A)** Whole mount view of male mammary ductal structures in wild type and *Edar^Tg951^*/*^951^* mice at 4, 6 and 8 weeks. Scale bar = 2 mm. **B)** Number of nipples present in adult female wild type and *Edar^Tg951^*/*^951^* mice (**** *P* <0.0001, N≥9).

**Supplementary Figure 5.*Edar* is highly expressed in mammary tumours from MMTV-Wnt1 mice**

**A)** *Edar* expression in a series of mouse mammary tumour models. **B)** Western blot detecting β-catenin protein in contralateral mammary gland and small intestine tissues taken from the *Edar^Tg951^*/*^951^* animals carrying mammary tumours T130314 and T010414. The shortened form is only produced by the two tumour samples.

**Supplementary Figure 6. *Edar* expression and gland histology through pregnancy.**

**A)** qRT-PCR showing *Edar* expression at different stages of the second pregnancy cycle in wild type and *Edar^Tg951^*/*^951^* glands (*** *P* <0.001, N=3). **B)** In situ hybridisation detecting *Edar* expression at pregnancy day 18 (P18). Scale bar = 50 μm. C) Histological structure of wild type and *Edar^Tg951^*/*^951^*glands through the pregnancy cycle. Scale bar = 100 μm. **D-E)** Quantification of the proportion of epithelial area apparent on sections of wild type and *Edar^Tg951^*/*^951^*glands at involution day 6 in first and second pregnancy (N≥3).

**Supplementary Figure 7. EDAR signalling suppresses, but EDAR induced tumours rely on, β-catenin activity.**

**A)** β-catenin signalling activity in 293T cells transfected to express EDAR or EDAR with Δ exon 3 β-catenin. EDAR suppresses β-catenin activity, but the Δ exon 3 β-catenin expressing cells are resistant to this inhibition (**** *P* <0.0001, N=5). **B)** Cells from *Edar^Tg951^*/*^951^* normal mammary gland and tumour display high levels of the β-catenin target gene *Lef1*, the expression of which is suppressed by iCRT-3 treatment (N=1). **C)** Treating with iCRT-3 does not alter the incidence of apoptosis in cells isolated from *Edar^Tg951^*/*^951^* tumours. Apoptotic cells were detected by cleaved-Caspase-3 staining (N=3).

**Supplementary Figure 8. EDAR and Δ exon 3 β-catenin** **protein** **expression and signalling activity in EpH4 cells.**

**A)** Detection of EDAR protein by Western blot (Licor) in transiently transfected 293T and stably transduced EpH4 cells. **B)** Induction of EDAR target genes by transduction of EpH4 cells with a lentivirus encoding *Edar* (N=3). **C)** Detection of Δ exon 3 β-catenin protein by Western blot (Licor) in transiently transfected 293T and stably transduced EpH4 cells. **D)** Induction of the β-catenin target gene *Axin2* by expression of Δ exon 3 β-catenin in EpH4 cells (* *P* <0.05, N=3).

**Supplementary Tables**

| **Antigen** | **Source** | **Catalogue number** | **Dilution** |
| --- | --- | --- | --- |
| **Immunohistochemistry:** |  |  |  |
| Ki67 | ACRIS | DRM004 | 1:100 |
| Cleaved Caspase-3 | Cell Signalling | 9661 | 1:200 |
| Loricrin | AbCam | AB24722 | 1:500 |
| Keratin-1 | Abcam | AB24643 | 1:200 |
| Keratin-14 | Covance | PRB-155P | 1:200 |
| Keratin-18 |  |  |  |
| β‑catenin | BD Transduction labs | 610154 | 1:200 |
| Phospho-γH2AX | Abcam | AB11174 | 1:200 |
| **Western blotting:** |  |  |  |
| ER | Santa Cruz | Sc542 | 1:200 |
| α-tubulin | Abcam | AB18251 | 1:200 |
| Lamin B1 | Abcam | Ab16048 | 1:2000 |
| Myc epitope | Chemicon | MAB4410 | 1:1000 |
| p65 | Cell Signaling | 4764 | 1:1000 |

**Supplementary Table 1.** Primary antibodies used.

| **Target** | **Forward sequence 5’-3’** | **Reverse sequence 5’-3’** |
| --- | --- | --- |
| **qPCR** |  |  |
| *Edar* | CCAGGGAGAAGTCTGTGACC | CTGAGCATTCGGCTTGTCTT |
| *Axin2* | CACGTAGGTTCCGGCTATGT | CTGCGATGCATCTCTCTCTG |
| *Lef1* | TGAGTGCACGCTAAAGGAGA | ATAATTGTCTCGCGCTGACC |
| *Wnt10b* | GTTCACGAGTGTCAGCACCA | TTGCTCACCACTACCCTTCC |
| *Dkk4* | CTGGTGACCTTGCTTGGACT | CTCTGACACCTCCTGCGAAC |
| *A20/TNFAIP3* | CAGGGACAAGCAAGTGCAGG | CGGGGAACCAGCTTTCTCAG |
| *Keratin-18* | CGGGAACATCTGGAGAAGAA | CATCTACCACCTTGCGGAGT |
| **Sequencing (cDNA)** |  |  |
| *β-catenin 42-458* | TACCTGAAGCTCAGCGCACAGC | GCGCTGGACATTAGTGGGATGAG |
| Nested sequencing primers |  |  |
| *βcatenin 80-99* | CGTGGACAATGGCTACTCAA |  |
| *βcatenin 301-320* |  | TGCTCTTGCGTGAAGGACTG |
| **Sequencing (genomic)** |  |  |
| β-catenin 16645-17847 | TATTTTTAGTGTATGCCATGGTGAACTGG | GCACCATTAGTTTCCAAAACTTAGTGTGA |
| Nested sequencing primers |  |  |
| mβcatenin 16722 | GACCTGGGTTAAAAACAAACTTAAG |  |
| mβcatenin 17750 |  | GCAAGTTACATCACTGCTTA |

**Supplementary table 2.** Primers used
