# Supplementary figures and images for: Elevated EDAR signalling promotes mammary gland tumourigenesis with squamous metaplasia
Source: Oncogene. 2021 Dec 16;41(7):1040–9. doi: 10.1038/s41388-021-01902-6 (PMC8837535; doi:10.1038/s41388-021-01902-6)

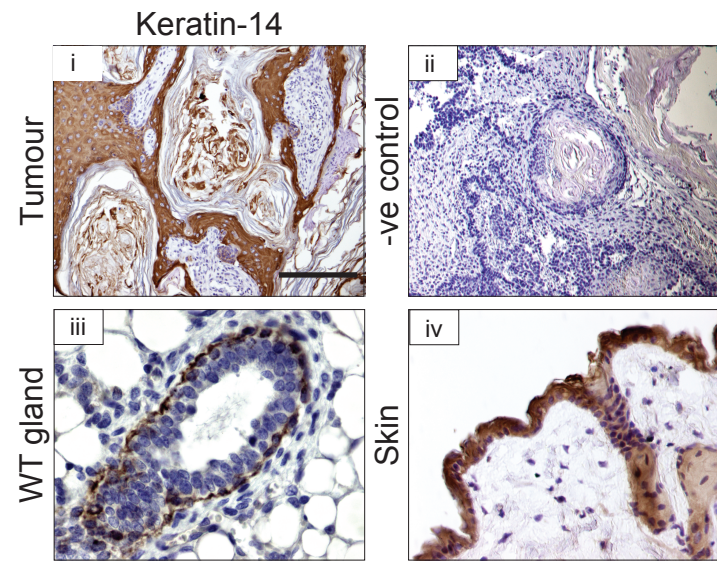

Supplement: Supplementary file 2 — Supplementary Figure 1 [file 41388_2021_1902_MOESM2_ESM.pdf]

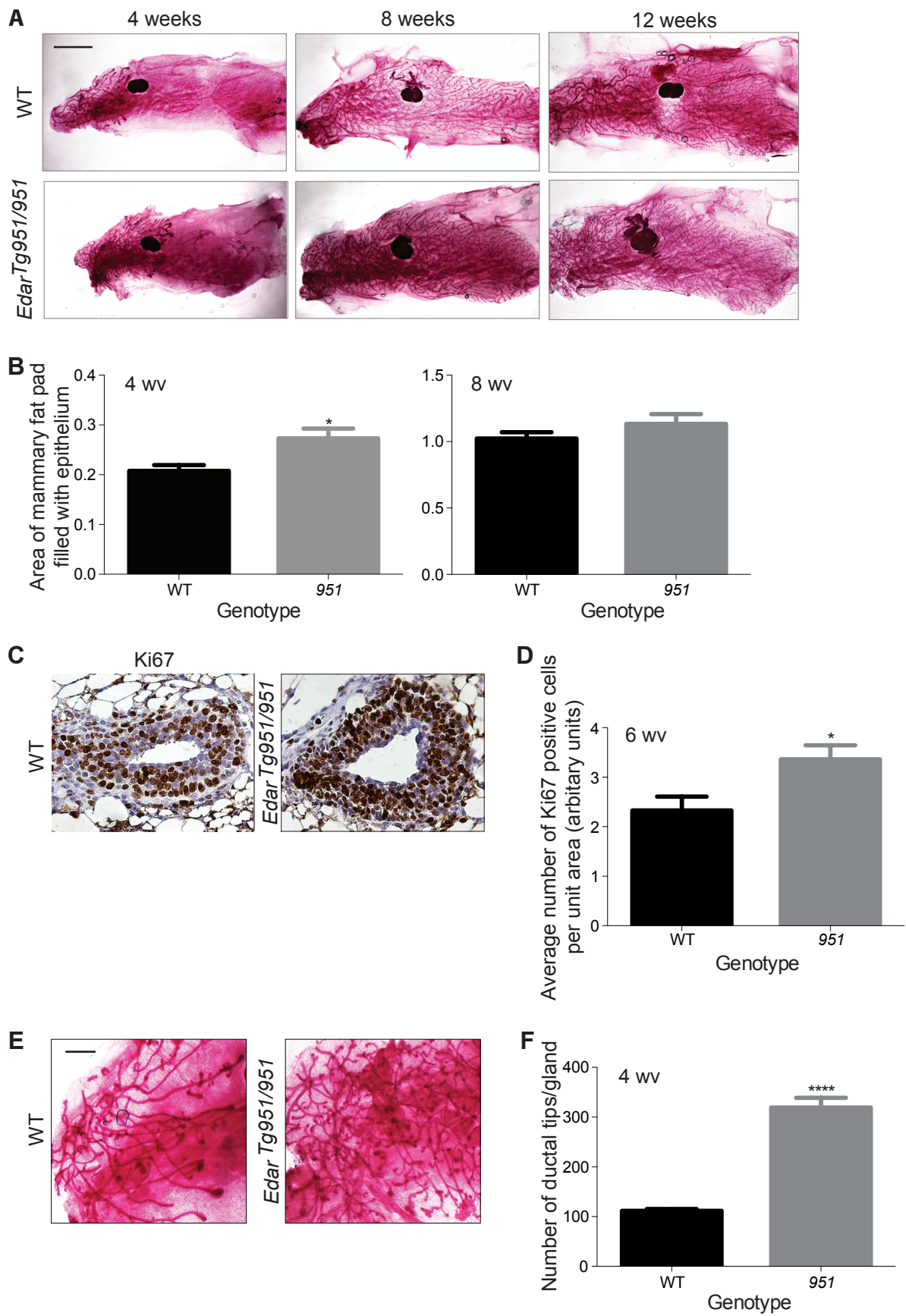

Supplement: Supplementary file 3 — Supplementary Figure 2 [file 41388_2021_1902_MOESM3_ESM.pdf]

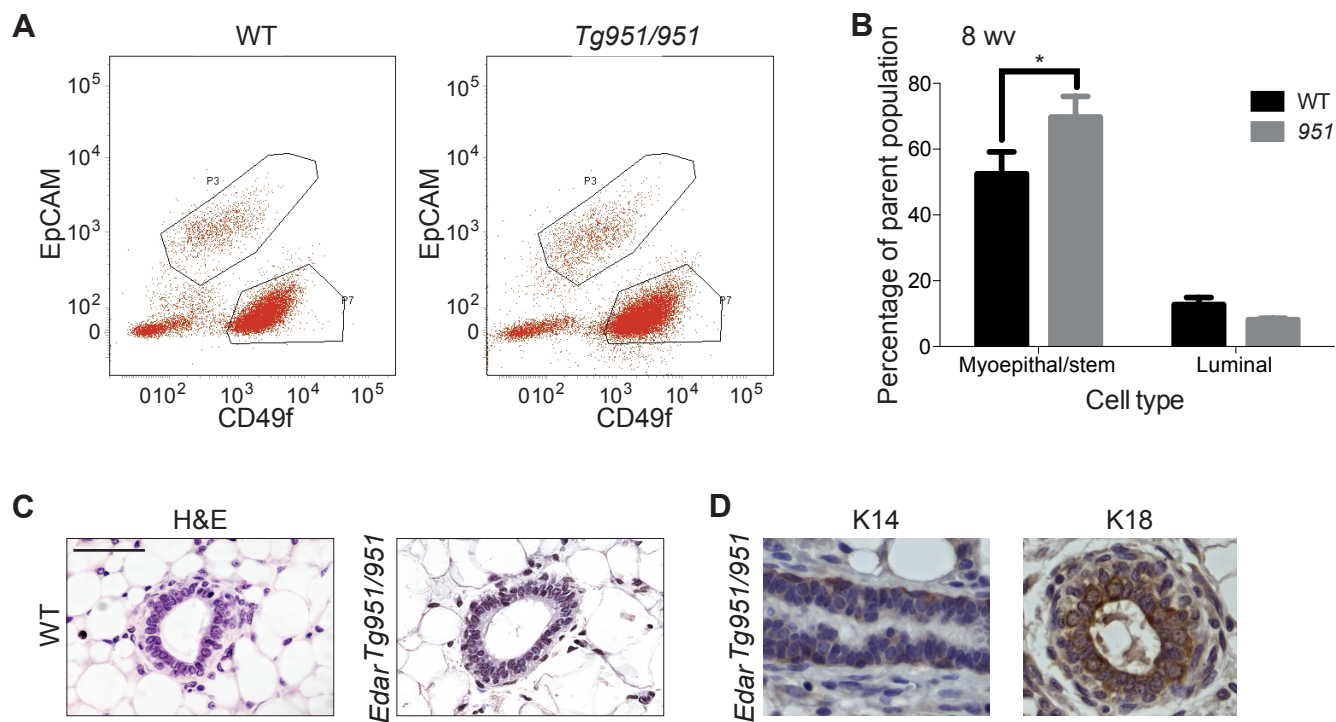

Supplement: Supplementary file 4 — Supplementary Figure 3 [file 41388_2021_1902_MOESM4_ESM.pdf]

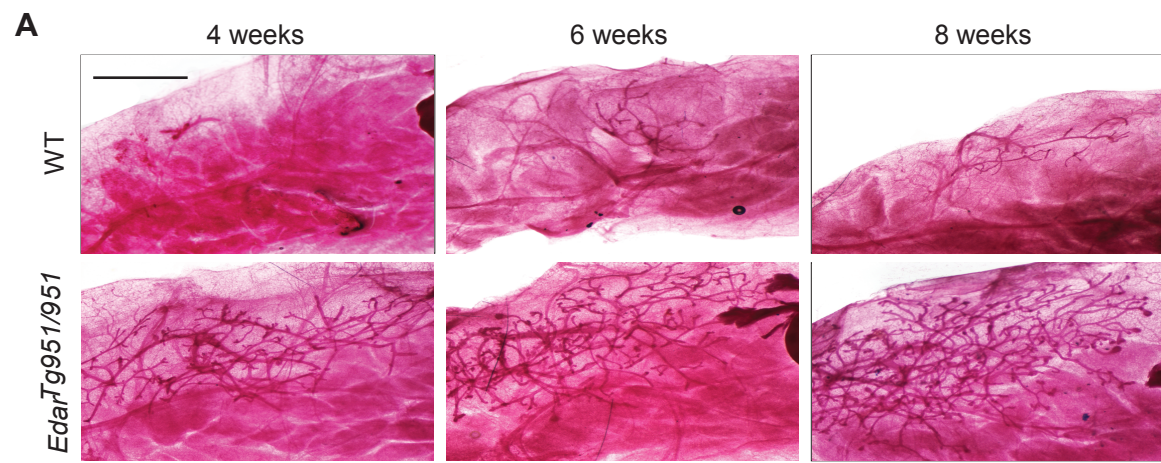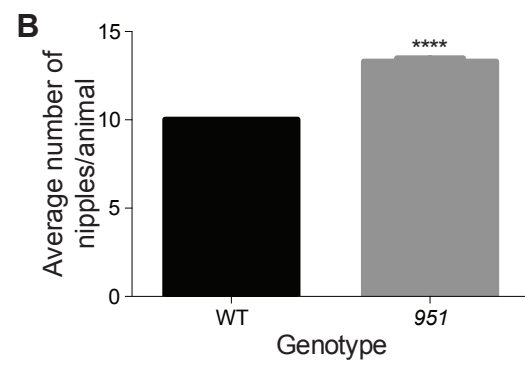

Supplement: Supplementary file 5 — Supplementary Figure 4 [file 41388_2021_1902_MOESM5_ESM.pdf]

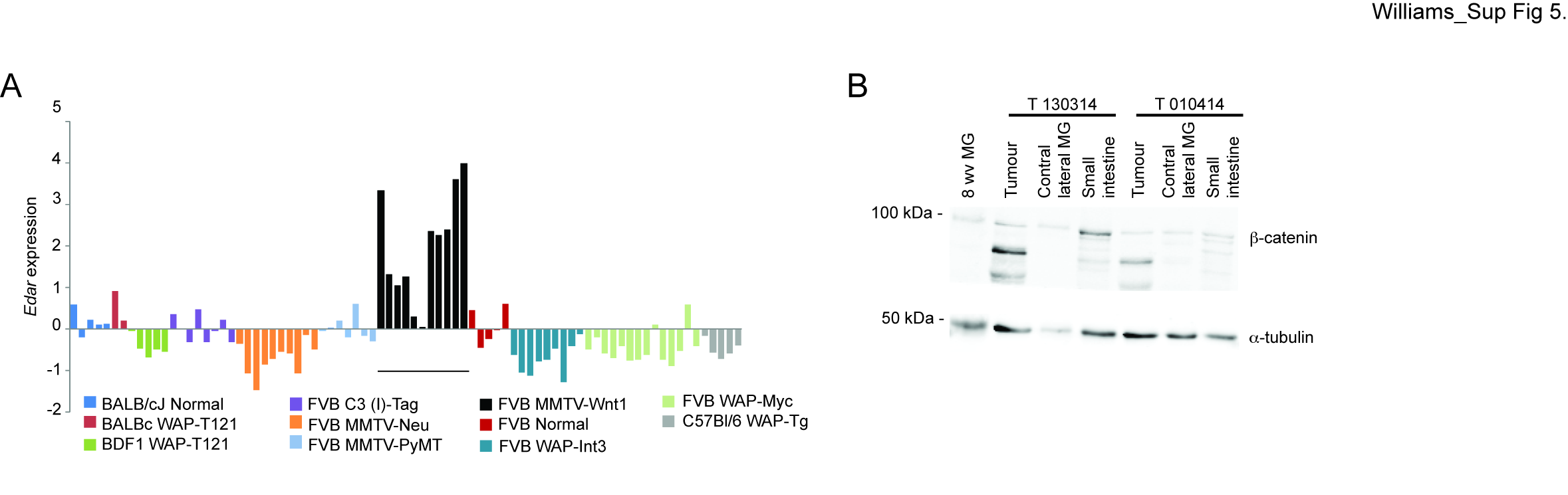

Supplement: Supplementary file 6 — Supplementary Figure 5 [file 41388_2021_1902_MOESM6_ESM.tif]

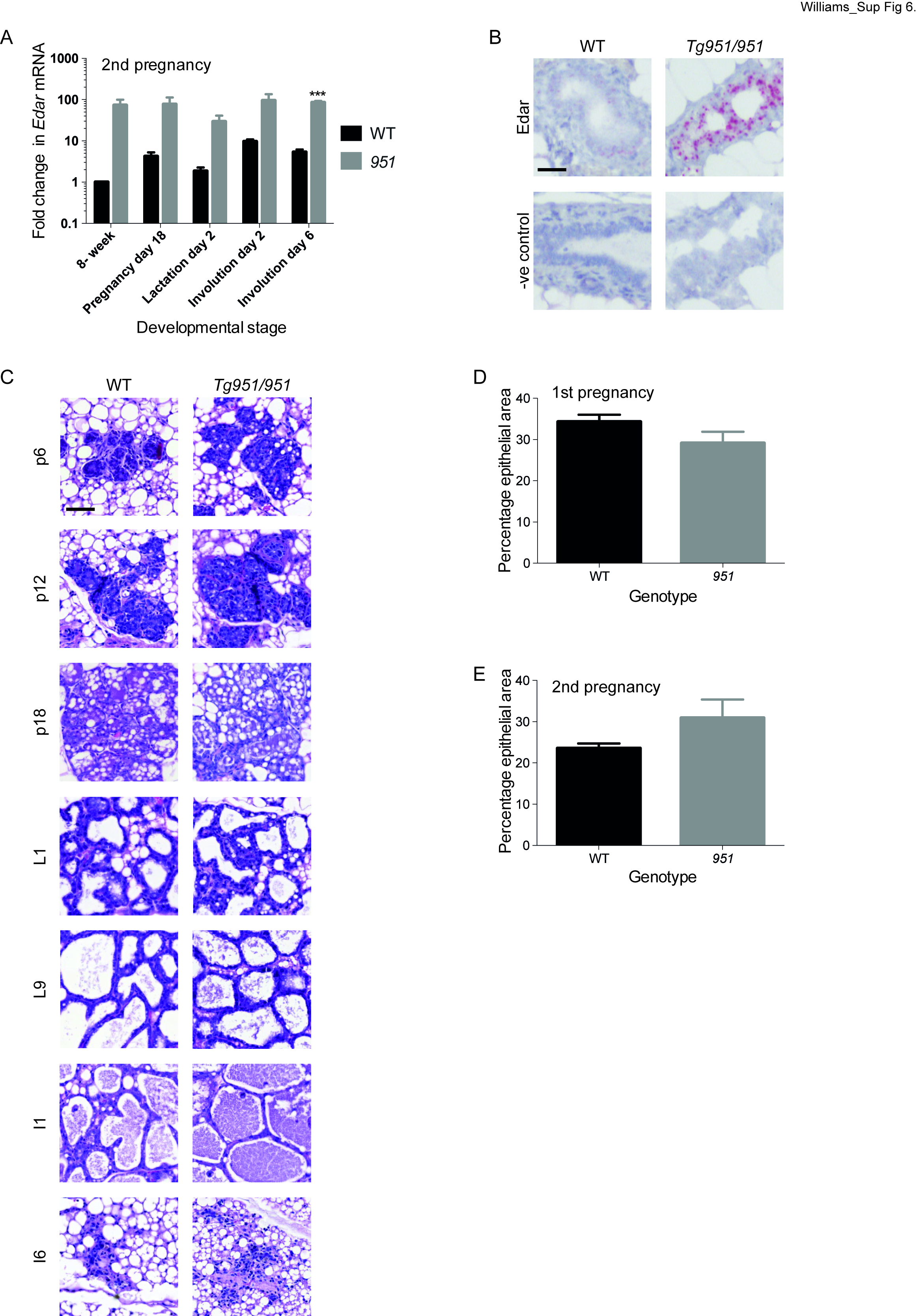

Supplement: Supplementary file 7 — Supplementary Figure 6 [file 41388_2021_1902_MOESM7_ESM.tif]

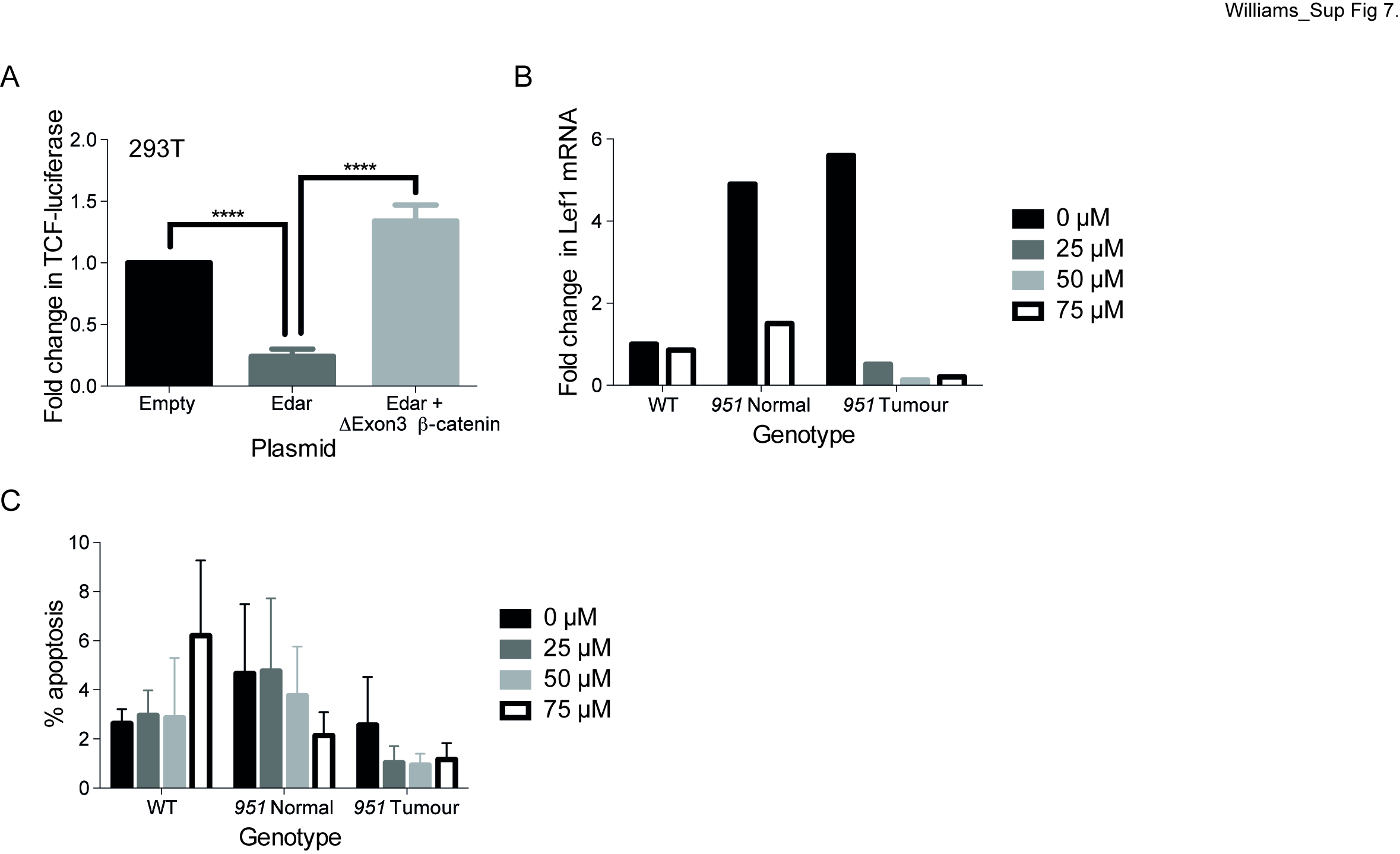

Supplement: Supplementary file 8 — Supplementary Figure 7 [file 41388_2021_1902_MOESM8_ESM.tif]

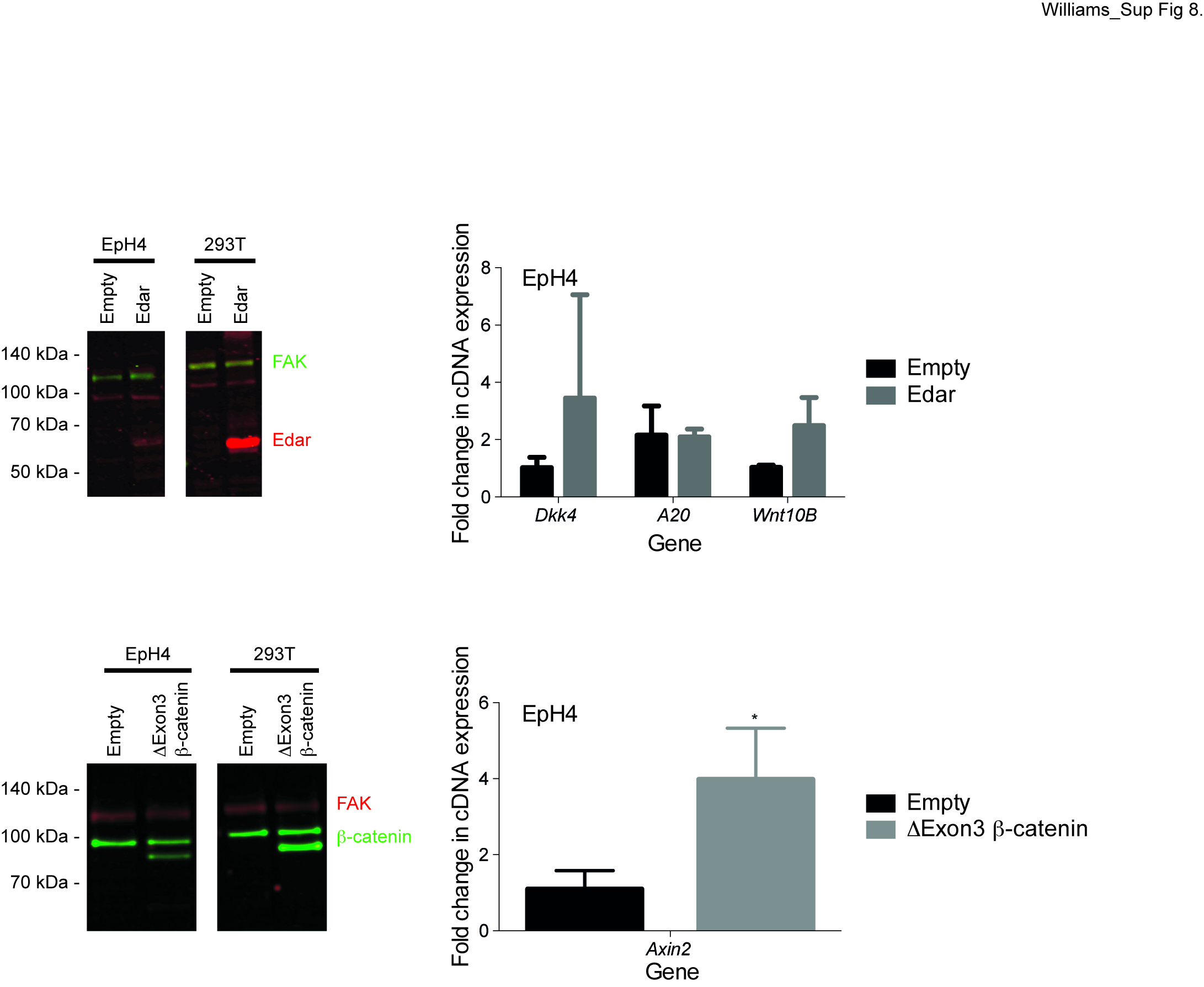

Supplement: Supplementary file 9 — Supplementary Figure 8 [file 41388_2021_1902_MOESM9_ESM.tif]
